# A comprehensive analysis and experimental validation of TK1 in uterine corpus endometrial carcinoma

**DOI:** 10.1038/s41598-024-56676-0

**Published:** 2024-03-13

**Authors:** Yiqing Sun, Kaiwen Zhang, Tianqi Wang, Shuangshuang Zhao, Chao Gao, Fengxia Xue, Yingmei Wang

**Affiliations:** 1 Department of Gynecology and Obstetrics, Tianjin Medical University General Hospital, Tianjin, 300052 China; 2 Tianjin Key Laboratory of Female Reproductive Health and Eugenics, Tianjin Medical University General Hospital, Tianjin, 300052 China

**Keywords:** TK1, UCEC, Prognosis, Diagnosis, Immune, Cancer genomics, Endometrial cancer, Cancer, Gene expression, Biomarkers, Endometrial cancer, Endometrial cancer

## Abstract

Uterine corpus endometrial carcinoma (UCEC) is becoming a main malignant cancer that threaten to women's health. Thymidine kinase 1 (TK1) is considering to be associated with tumorigenesis and development. Nevertheless, the function of TK1 in UCEC is still unclear. Herein, we analyzed the TK1 expression level in pan-cancer and found that TK1 was upregulated in a variety of cancers including UCEC. Patients of UCEC with high expression of TK1 were related to poor outcome. TK1 was also related to clinical stage, histologic grade and lymph node metastasis. Abnormal expression of TK1 in UCEC was related to promoter methylation while gene mutation was not frequent. TK1 and its associated genes appeared to be prominent in cell cycle and DNA replication, according to GO and KEGG analysis. Analysis of immune infiltration revealed a negative correlation between TK1 and CD8 + T cells, macrophages, and dendritic cells. In vitro experiments, TK1 knockdown resulted in the inhibition of proliferation, migration, invasion and EMT in UCEC cell lines.

## Introduction

Malignant tumors have become one of the major diseases that seriously threaten the health of global population^1^. In malignant neoplasms of the female reproductive system, due to widespread screening and the spread of cervical cancer (CC) vaccines, the incidence of CC has decreased significantly. Meanwhile, the incidence and mortality rate of ovarian cancer (OC) were both declined. In the USA, the incidence of uterine corpus endometrial carcinoma (UCEC) is higher than CC and OC, and become the fourth most commonly diagnosed cancer in women (after breast cancer, lung cancer and colorectal cancer). While the survival rate didn’t improved, the mortality rate in UCEC continue to increase about 1% per year^2^, reflects the lag in the diagnosis and treatment of UCEC.

During the 1960s, Thymidine kinase 1 (TK1) was discovered, a gene that mapped to chromosome 17q25.3 and encoded a protein with 25kDa^3^. TK1 located in cytoplasm and was a key enzyme for converting thymidine to thymine monophosphate, which was essential for DNA synthesis^3,4^. The level of TK1 was highest in S phase and closely related to cell cycle^5^. While another isoenzyme called TK2 located in mitochondria and involved in mitochondrial DNA replication rather than cell cycle^6^. TK1 has been found to have elevated levels in a variety of tumors. A systematic meta-analysis indicated that overexpression of TK1 was associated with poor outcomes in lung cancer^7^. A study containing 56,178 people revealed that serum TK1 (sTK1) might be a potential marker for predict persons in the risk of pre-cancer or already trapped in cancer^8^. TK1 might be a potential marker for monitoring the treatment response and prognosis^9^.

But the value of TK1 in UCEC is still unclear. In this paper, we clarified the TK1 expression in UCEC, revealed its mutation and methylation, to show its great significance in diagnosis and prognosis.

## Results

### Identification of TK1 in pan-cancer and UCEC

**Figure 1:**
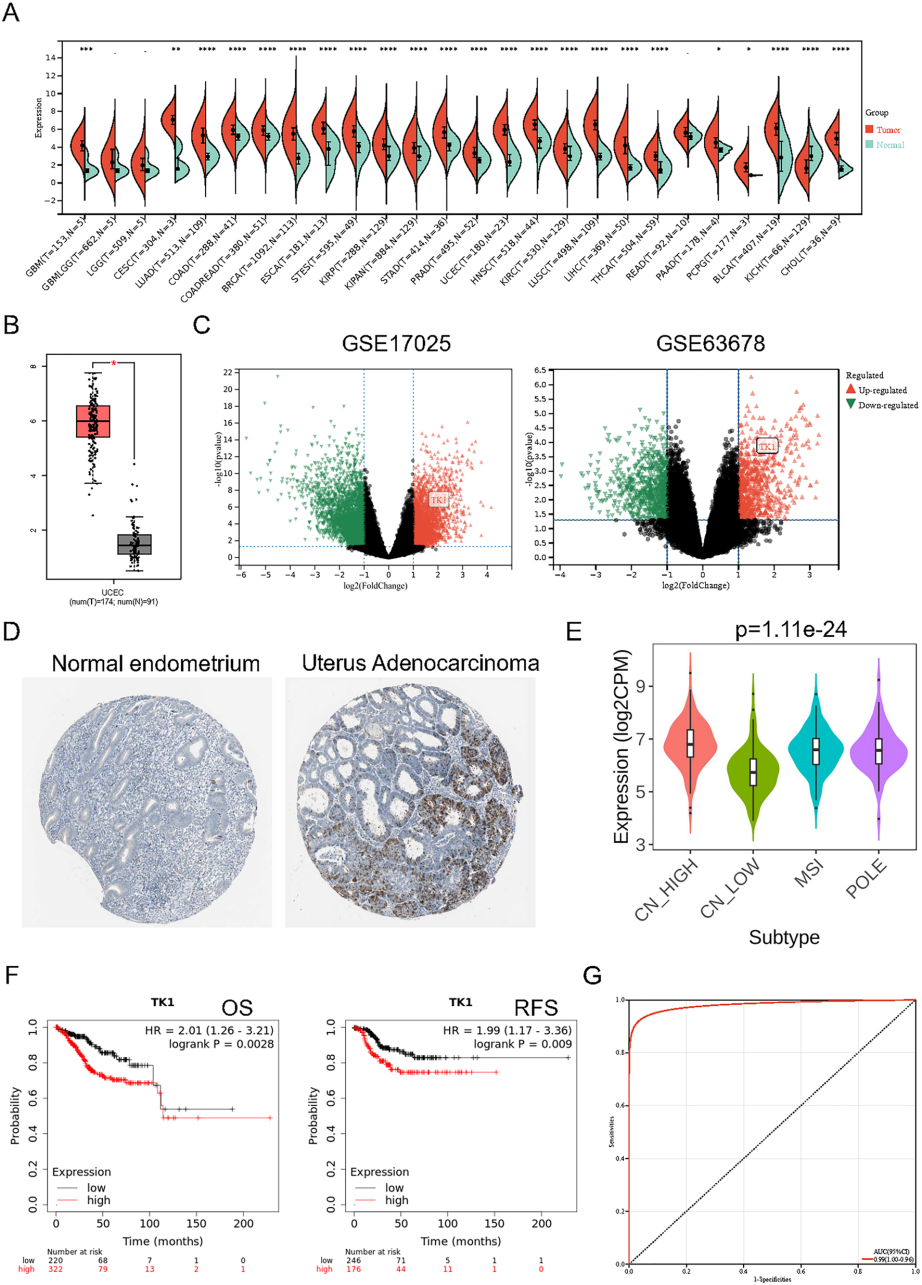
Expression and prognostic value of TK1 in UCEC. (**A**) Expression of TK1 in pan-cancer. (**B**) TK1 expression in UCEC based on TCGA and GTEx data. (**C**) TK1 expression in GSE17025 and GSE63678. (**D**) IHC of TK1 in normal endometrium and UCEC. (**E**) The relationship between TK1 expression and molecular type. (**F**) The prognostic value to predict OS and RFS of TK1 in UCEC. (**G**) The diagnostic value of TK1 in UCEC.

A unified and standardized pan-cancer dataset was downloaded from the UCSC (https://xenabrowser.net/) database. Then the expression of TK1 in each sample was extracted and transformed expression value using log2 (X + 1). Cancers that less than 3 samples were eliminated and finally obtained the expression data of 26 cancer types. The level of TK1 expression was upregulated in 25 cancers except Kidney Chromophobe (KICH). Furthermore, the expression of TK1 in 22 cancer tissues showed significant difference compared with normal tissue, including Glioblastoma multiforme (GBM), Cervical squamous cell carcinoma and endocervical adenocarcinoma (CESC), Lung adenocarcinoma (LUAD), Colon adenocarcinoma (COAD), Colon adenocarcinoma/Rectum adenocarcinoma Esophageal carcinoma (COADREAD), Breast invasive carcinoma (BRCA), Esophageal carcinoma (ESCA), Stomach and Esophageal carcinoma (STES), Kidney renal papillary cell carcinoma (KIRP), Pan-kidney cohort (KIPAN), Stomach adenocarcinoma (STAD), Prostate adenocarcinoma (PRAD), Uterine Corpus Endometrial Carcinoma (UCEC), Head and Neck squamous cell carcinoma (HNSC), Kidney renal clear cell carcinoma (KIRC), Lung squamous cell carcinoma (LUSC), Liver hepatocellular carcinoma (LIHC), Thyroid carcinoma (THCA), Prostate adenocarcinoma (PAAD), Pheochromocytoma and Paraganglioma (PCPG), Bladder Urothelial Carcinoma (BLCA), Cholangiocarcinoma (CHOL) (Fig. 1A). In order to clarify the TK1 expression in UCEC, GEPIA was used and it showed that TK1 was significantly overexpression in TCGA-UCEC (*p* < 0.05) compared with TCGA normal and GTEx data (Fig. 1B). Moreover, GEO datasets including GSE17025 (log2FC = 1.62, *p* < 0.0001) and GSE63678 (log2FC = 1.51, *p* < 0.0001) displayed the same result (Fig. 1C). Immunohistochemistry (IHC) indicated that the stain of TK1 in UCEC was stronger than in normal endometrium. Besides, TK1 largely located in glandular cells rather than endometrial stroma, and in glandular cells it mostly located in cytoplasm (Fig. 1D). In addition, TK1 expression was also strongly associated with molecular subtype in UCEC (Fig. 1E). Considering that TK1 was upregulated in most cancers, whether it could predict prognosis was unclear. Thus, KM-plotter was used to analyze its value in UCEC. High level of TK1 in UCEC means worse overall survival (OS) (*p* = 0.0028) and recurrence free survival (RFS) (*p* = 0.009, Fig. 1F). Moreover, with an AUC of 0.99, TK1 expression might also be employed to separate UCEC tissues from normal tissues (Fig. 1G).

### The association between TK1 expression and UCEC clinical characteristics

In order to further explore the influence of TK1 expression on UCEC progression, TCGA-UCEC clinical dataset was used to evaluate the correlation between TK1 expression and clinical characteristics of UCEC. According to the median of TK1, samples were divided into two groups called high expression and low expression. Between the two groups, there were great significant difference in BMI, histological type, histological grade, clinical stage and lymph node metastasis. In contrast, age of onset, menopause status, tumor invasion and surgical approach seemed to be not related to TK1 expression (Table 1).

**Table 1:** The relationship between TK1 mRNA level and clinical characteristics of UCEC.

Clinical features	Number of cases	Low expression of TK1, number (%)	High expression of TK1, number (%)	*p*
Age				0.172
≤ 60	178	93 (52.25)	85 (47.75)	
> 60	362	167 (46.16)	195 (53.84)	
BMI				0.000
< 25	95	27 (28.42)	68 (71.58)	
≥ 25, < 30	114	57 (50.00)	57 (50.00)	
≥ 30	303	161 (53.14)	142 (46.86)	
Menopause status				0.268
Pre	35	21 (60.00)	14 (40.00)	
Peri	34	17 (50.00)	17 (50.00)	
Post	445	205 (46.07)	240 (53.93)	
Histological type				0.000
Endometrioid	407	224 (55.04)	183 (44.96)	
Mixed	22	8 (36.36)	14 (63.64)	
Serous	114	29 (25.44)	85 (74.56)	
Histologic grade				0.000
G1	98	73 (74.49)	25 (25.51)	
G2	120	72 (60.00)	48 (40.00)	
G3	325	116 (35.69)	209 (64.31)	
Tumor invasion (%)				0.126
< 50	259	135 (52.12)	124 (47.87)	
≥ 50	211	95 (45.02)	116 (54.98)	
Clinical stage				0.016
Stage I	339	179 (52.80)	160 (47.20)	
Stage II	51	22 (43.14)	29 (56.86)	
Stage III	124	52 (41.94)	72 (58.06)	
Stage IV	29	8 (27.60)	21 (72.41)	
Lymph node metastasis				0.012
No	406	202 (49.75)	204 (50.24)	
Yes	81	28 (34.57)	53 (65.43)	
Surgical approach				0.211
Minimally invasive	201	103 (51.24)	98 (48.76)	
Open	320	146 (45.63)	174 (54.38)	

### The methylation of TK1 in UCEC

**Figure 2:**
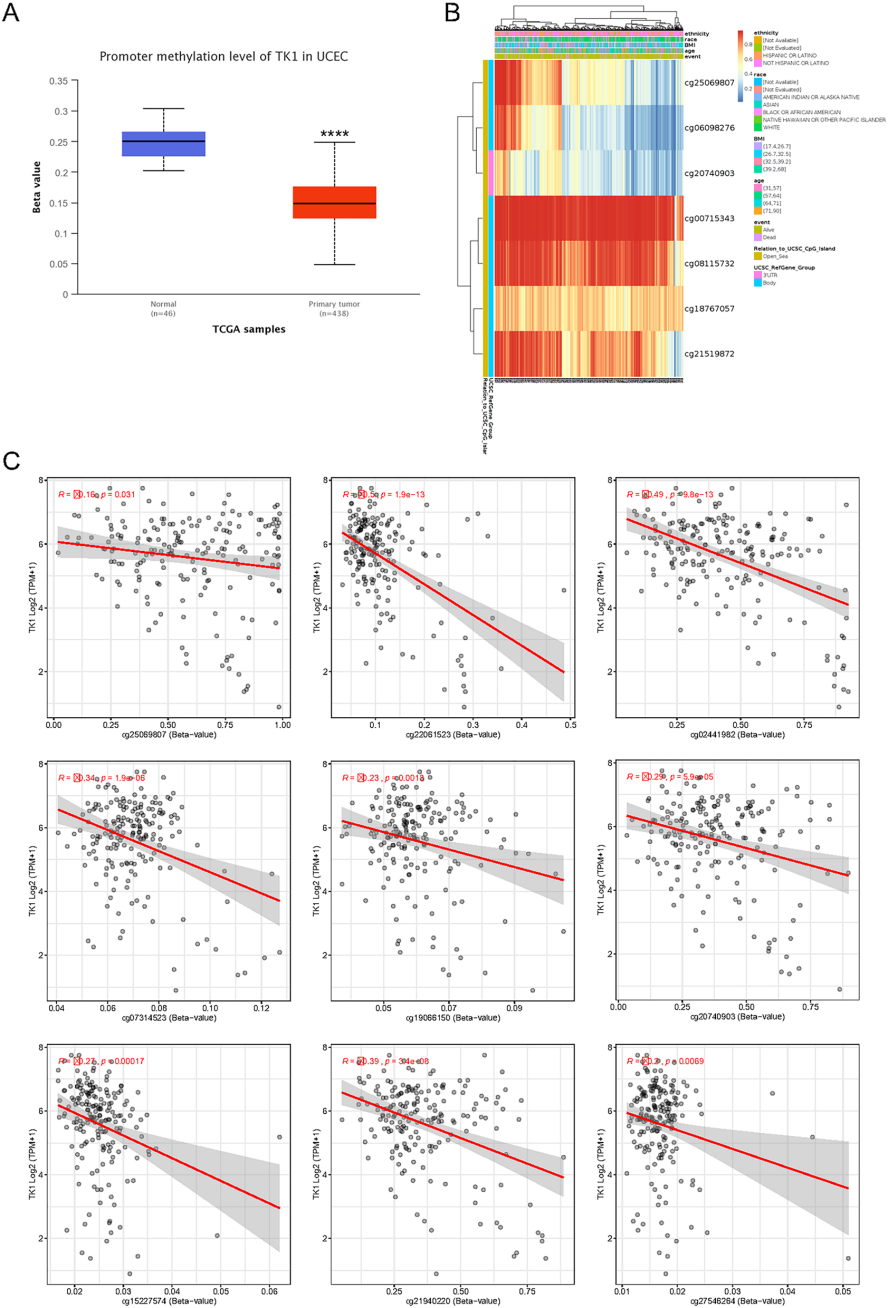
The methylation of TK1 in UCEC. (**A**) Total promoter methylation level of TK1 in UCEC. (**B**) The common methylation sites of TK1 in UCEC. (**C**) The relationship between methylation sites and TK1 expression.

Gene expression can be influenced by many processes, including modification after transcription, translation, epigenetic modify et al. Gene methylation is a part of epigenetic modify and negatively related to gene expression. In UCEC, total promoter region methylation level of TK1 was quietly lower than normal tissue suggesting that TK1 was an active condition in tumor tissues (Fig. 2A). Next, we analyzed TK1 methylation site, cg00715343 was the most common site (Fig. 2B). In addition, we explored the relationship between methylation sites and TK1 expression. Hypermethylation of cg25069807, cg22061523, cg02441982, cg07314523, cg19066150, cg20740903, cg15227574, cg21940220 and cg27546264 were negatively correlated with gene expression of TK1 (Fig. 2C). While hypermethylation of cg26206461, cg08115732, cg00715343 and cg21519872 were positively correlated with gene expression of TK1 (Supplementary Fig. 1A). Methylation of cg18767057, cg03291825, cg06098276, cg07379000 and cg20104688 didn’t show any significance with gene expression of TK1 (Supplementary Fig. 1B).

### The mutation status of TK1 in UCEC

**Figure 3:**
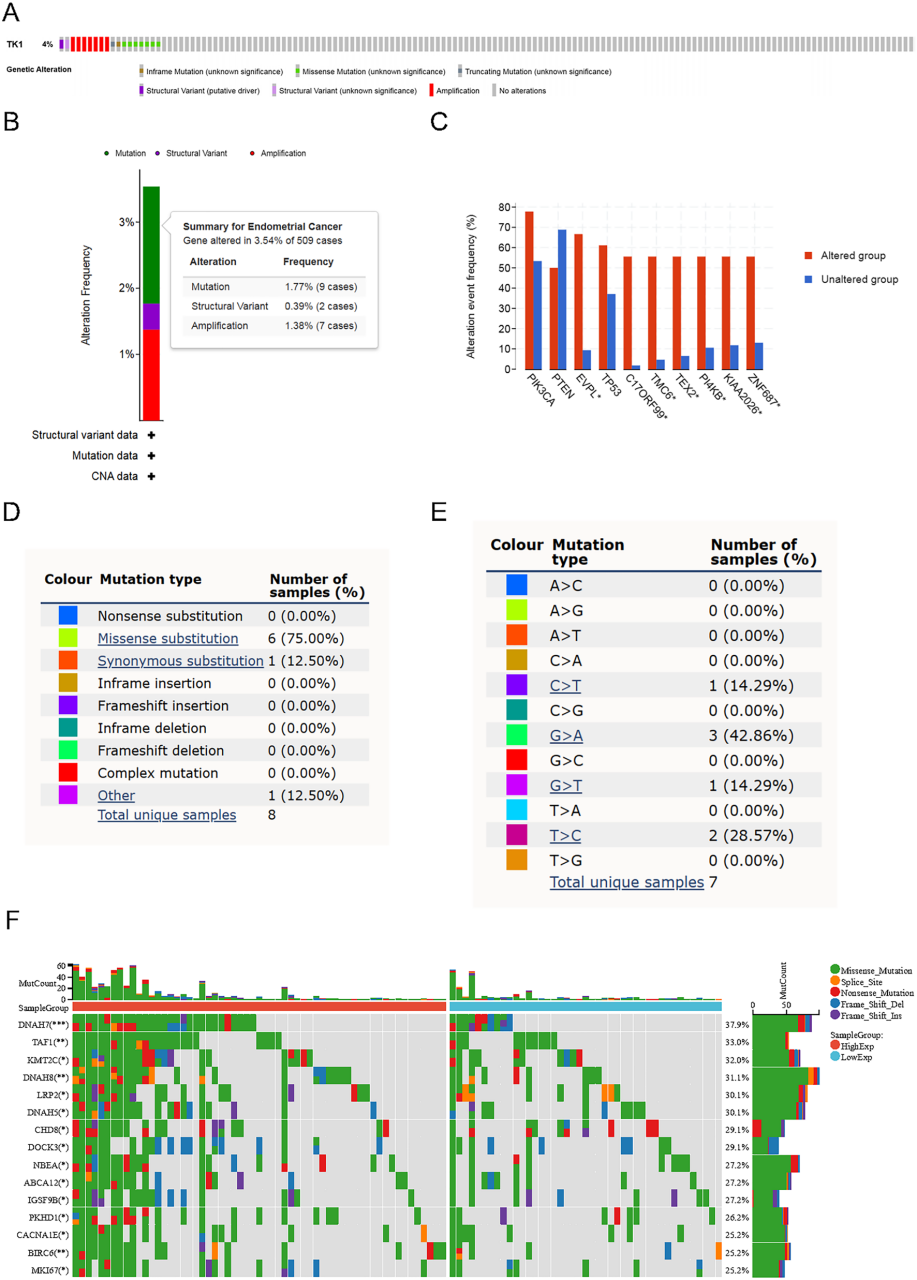
The mutation status of TK1 in UCEC. (**A** and **B**) Genetic alteration types and frequency of TK1 in UCEC. (**C**) Alteration event frequency between TK1 altered group and unaltered group. (**D** and **E**) The mutation types and base substitution of TK1 in UCEC. (**F**) Genetic mutation frequency between TK1 high expression group and low expression group.

Genetic alteration is consisted of inframe mutation, missense mutation, truncating mutation, structural variant and amplification. The mutation frequency of TK1 in UCEC was approximately 4%, with 1.77% mutation (especially missence substitution), 1.38% amplification and 0.39% structural variant (Fig. 3A,B,D). Genes in TK1 altered group including EVPL, TMC6, PI4KB, KIAA2026, ZNF687 showed highly alteration frequency. But there was no significance of PIK3CA, PTEN and TP53 (Fig. 3C). The majority base substitution in UCEC was G > A (42.86%), while others were T > C (28.57%), C > T (14.29%) and G > T (14.29%) (Fig. 3E). In TK1 high expression group, MKI67 mutation frequency was increased (Fig. 3F).

### The PPI network of TK1

**Figure 4:**
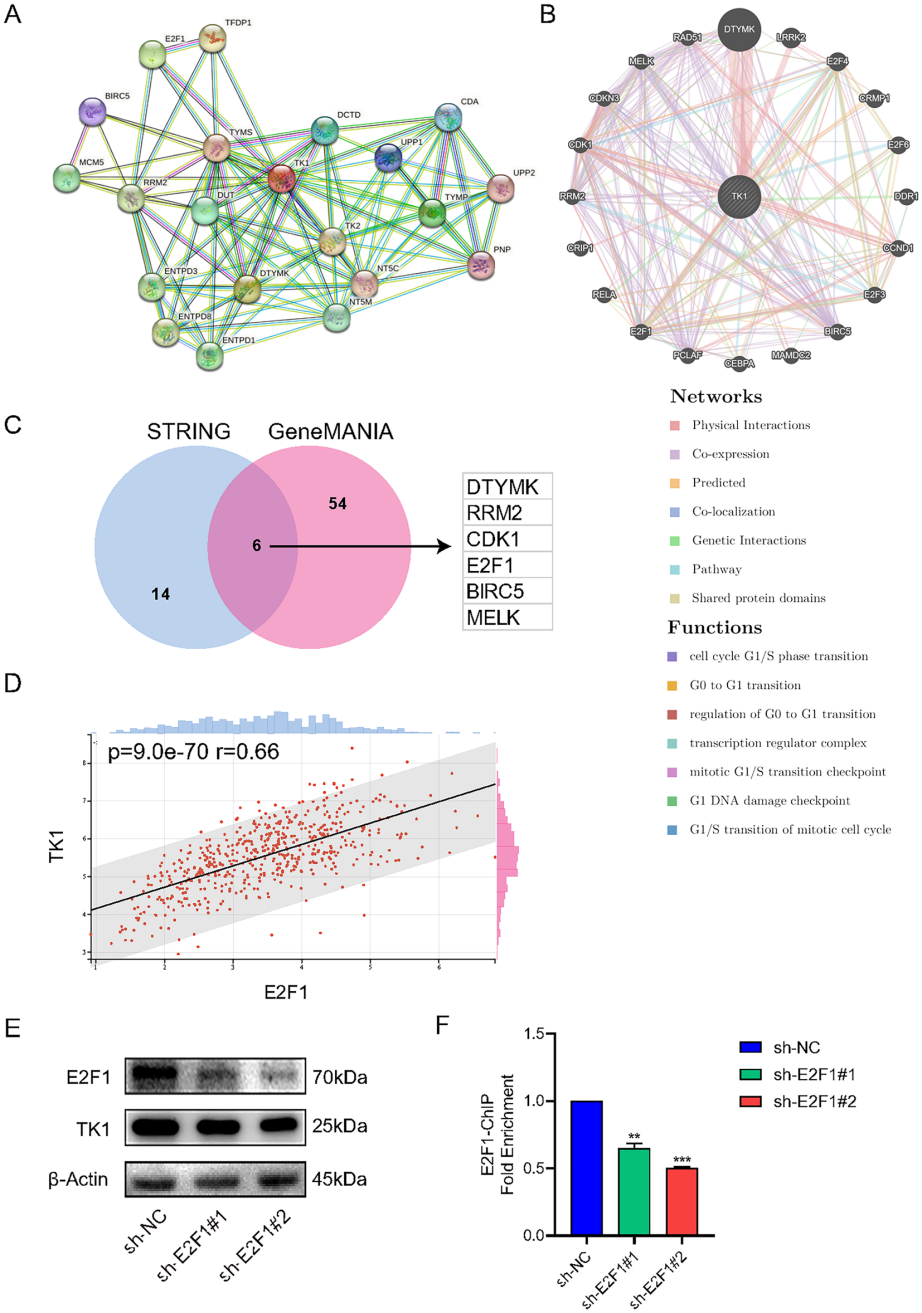
The PPI network of TK1. (**A**) TOP20 genes related to TK1 based on STRING database. (**B**) The associated genes with TK1 in GeneMANIA database. (**C**) The interaction of STRING and GeneMANIA with TK1 binding proteins, including DTYMK, RRM2, CDK1, E2F1, BIRC5, MELK. (**D**) The correlation between TK1 and E2F1. (**E**) After E2F1 knockdown in HEC-1B, TK1 expression was also decreased (blots were cut prior to hybridisation with antibodies during blotting). (**F**) ChIP at promotor regions of TK1 in HEC-1B after E2F1 knockdown.

TK1 might interact with many proteins or genes to make difference on tumorigenesis. It is important to clarify TK1-interacting proteins and their functions. STRING database was used to create PPI network, which provide interaction evidence by textmining, experiments, databases, co‑expression, neighborhood, gene fusion and co‑occurrence. With our criteria, 60 proteins were detected to have interaction with TK1 and TOP10 proteins were as follows: DUT, DTYMK, DCTD, CDA, UPP1, PNP, TYMP, TYMS, E2F1 and BIRC5 (Fig. 4A). Next GeneMANIA database was applied to protract GGI network, with 20 genes (DDR1, RELA, CCND1, E2F3, E2F6, MAMDC2, CRIP1, CRMP1, LRRK2, DTYMK, E2F4, RRM2, CDK1, RAD51, E2F1, BIRC5, PCLAF, CEBPA, MELK and CDKN3). The function mostly enriched in cell cycle (Fig. 4B). The common TK1-interacting proteins in both STRING and GeneMANIA were DTYMK, RRM2, CDK1, E2F1, BIRC5 and MELK (Fig. 4C). E2F1, a star transcription factor, binds to the promoter region of genes and participates in the regulation of gene expression^10^. Thus, E2F1 might be a transcription factor of TK1. We performed a correlation analysis of E2F1 and TK1 and found a positive correlation (Fig. 4D). To clarify the relationship between E2F1 and TK1, we transfected sh-E2F1 into HEC-1B and then detect the expression of TK1. In our results, E2F1 knockdown could downregulate TK1 expression (Fig. 4E). ChIP assay demonstrated that E2F1 combined with the promotor region of TK1 and regulated its expression (Fig. 4F).

### Enrichment analysis of TK1 in UCEC

**Figure 5:**
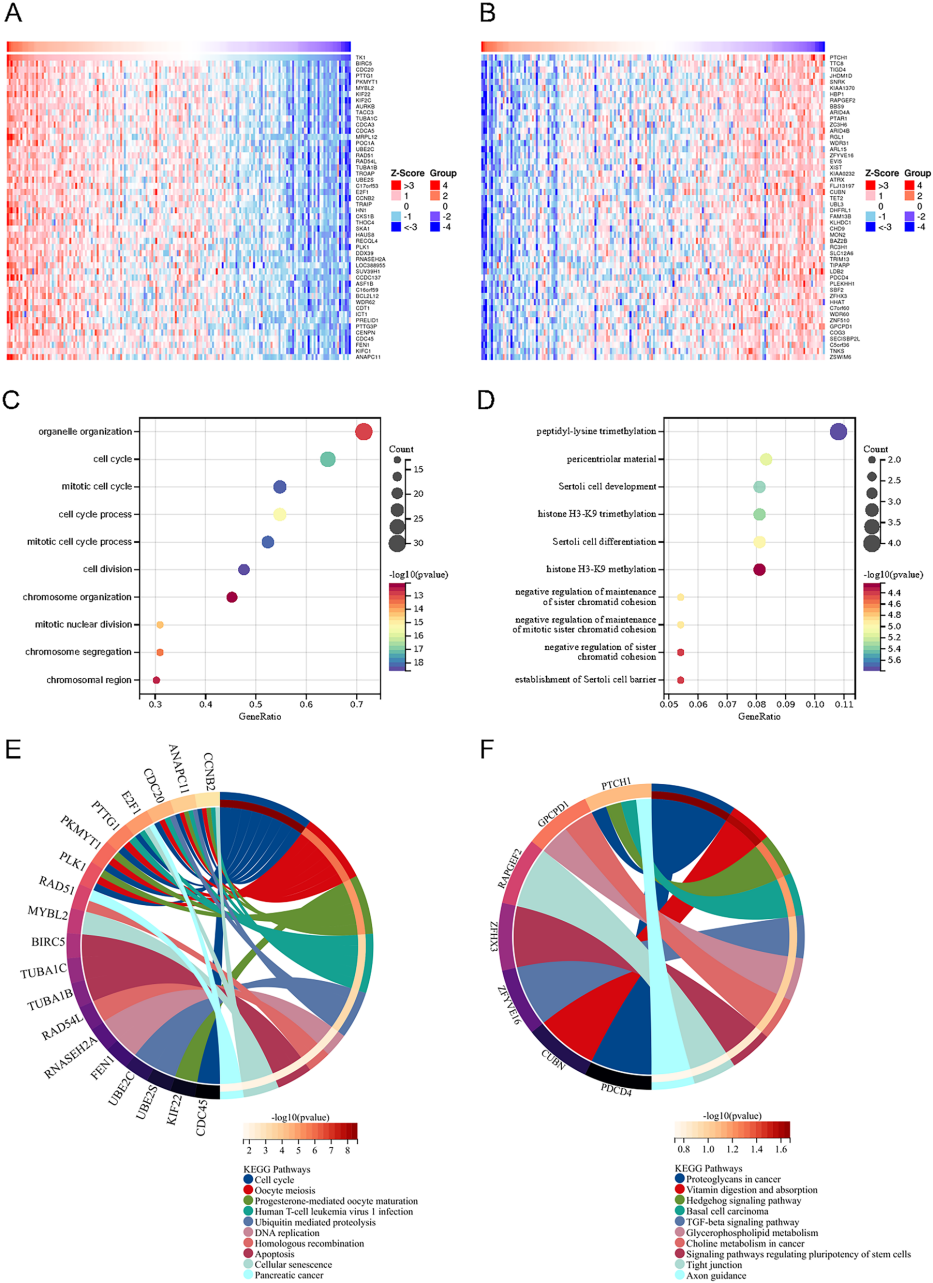
Gene functional analyses of TK1 in UCEC. TOP50 genes that positively (**A**) and negatively (**B**) related to TK1 in UCEC. GO enrichment analysis in TOP50 TK1 positively (**C**) and negatively (**D**) related genes. KEGG enrichment analysis in TOP50 TK1 positively (**E**) and negatively (**F**) related genes.

TK1-related genes were explored by LinkedOmics portal. We listed TOP50 genes that positively related to TK1 (Fig. 5A) and TOP50 genes negatively related to TK1 (Fig. 5B). Then GO and KEGG enrichment analysis were completed using the two group genes. In GO enrichment analysis, TOP50 genes positively related to TK1 were enriched in organelle organization, cell cycle and cell division (Fig. 5C). While TOP50 genes negatively related to TK1 were enriched in peptidyl-lysine trimethylation, histone H3-K9 trimethylation and negative regulation of maintenance of mitotic sister chromatid cohesion (Fig. 5D). In KEGG enrichment analysis, TOP50 genes positively related to TK1 were enriched in cell cycle, ubiquitin mediated proteolysis, DNA replication and apoptosis (Fig. 5E). While TOP50 genes negatively related to TK1 were enriched in proteoglycans in cancer, Hedgehog signaling pathway and TGF-beta signaling pathway (Fig. 5F).

### The association of immune cell infiltration and TK1 in UCEC

**Figure 6:**
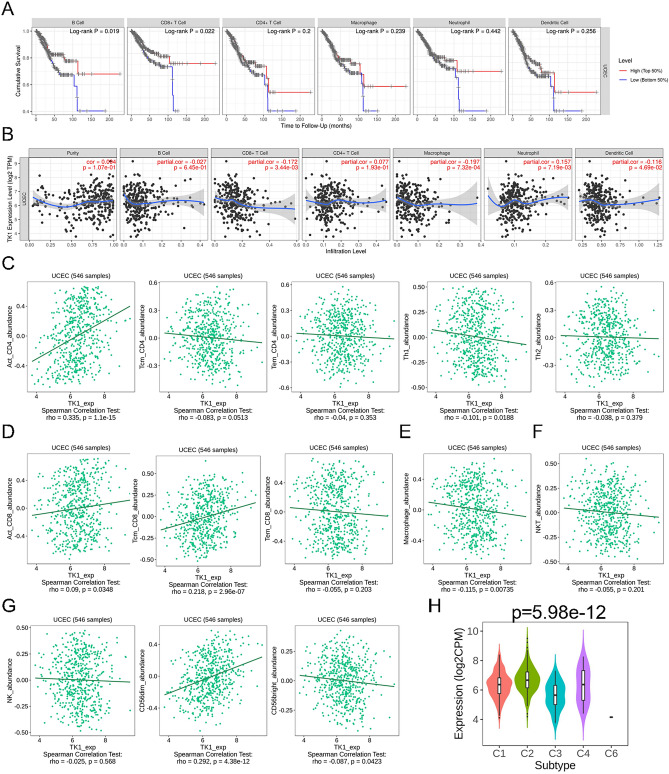
The correlation of TK1 expression and immune cell infiltration. (**A**) The association between immune cell infiltration and prognosis in UCEC. (**B**) The relationship between TK1 mRNA expression and immune cell infiltration level. (**C**) The correlation between different CD4 + T cell abundance and TK1 expression in UCEC. (**D**) The correlation between different CD8 + T cell abundance and TK1 expression in UCEC. (**E**) The correlation between macrophage abundance and TK1 expression in UCEC. (**F**) The correlation between NKT cell abundance and TK1 expression in UCEC. (**G**) The correlation between different NK cell abundance and TK1 expression in UCEC. (**H**) The relationship between TK1 expression and immune subtypes.

Tumor immunity is becoming a new treatment direction. Immune cell infiltration is closely related to treatment efficacy and patient outcomes. It is important to know immune cell infiltration in tumor. TIMER database was applied on clarify the correlation between immune cell infiltration and prognosis in UCEC patients. B cell (*p* = 0.019) and CD8 + T cell (*p* = 0.022) were closely related to the prognosis in UCEC patients while CD4 + T cell, macrophage, neutrophil, and dendritic cell showed no significance (Fig. 6A). Next, we analyzed the relationship between TK1 and immune cell infiltration in UCEC. TK1 expression was positively related to neutrophil (r = 0.157, *p* = 7.19e−03) while negatively related to CD8 + T cell (r = − 0.172, *p* = 3.44e−03), macrophage (r = − 0.197, *p* = 7.32e−04) and dendritic cell (r = − 0.116, *p* = 4.96e−02) (Fig. 6B). Immune cells contain many subtypes. TISDIB database was used to investigate the relationship between TK1 and immune cell subtypes. In the CD4 + T cell subtype, TK1 was positively related to activated CD4 + T cell (r = 0.335, *p* = 1.1e−15) and negatively related to Th1 cell (r = − 0.101, *p* = 0.0188) (Fig. 6C). The abundance of activated CD8 + T cell (r = 0.09, *p* = 0.0348) and central memory CD8 + T cell (r = 0.218, *p* = 2.96e−07) were both positively associated with TK1 expression (Fig. 6D). Macrophage abundance (r = − 0.115, *p* = 0.00735) was negatively correlated with TK1 expression (Fig. 6E). Natural killer T cell (NKT cells) is a special subset of T cells with both T cell receptor and NK cell receptor on the cell surface. There was a negative correlation between NKT cell abundance and TK1 expression while with no great significance (Fig. 6F). Natural killer cell (NK cell) is a unique group of innate immune lymphocytes with innate cytotoxicity and immunomodulatory abilities. It mainly divides into two groups called CD56 bright subtype and CD56 dim subtype. CD56 bright is less common in peripheral blood, estimated to make up only 5–10% of total NK cells, while CD56 dim NK cells make up more than 90%. However, CD56 bright is rich in tissues, including secondary lymphoid tissues. Results revealed that although NK cell abundance (r = − 0.025, *p* = 0.568) showed no significance with TK1 expression, but CD56 dim abundance (r = 0.292, *p* = 4.38e−12) was positively associated with TK1 expression and CD56 bright abundance (r = − 0.087, *p* = 0.0423) was negatively associated with TK1 expression (Fig. 6G). UCEC was also divided into six immune classifications, including wound healing (C1), IFN-gamma dominant (C2), inflammatory (C3), lymphocyte depleted (C4), immunologically quiet (C5), TGF-β dominant (C6). TK1 expression was significantly associated with those six immune subtypes (Fig. 6H).

### Knockdown TK1 inhibited proliferation in UCEC cells

**Figure 7:**
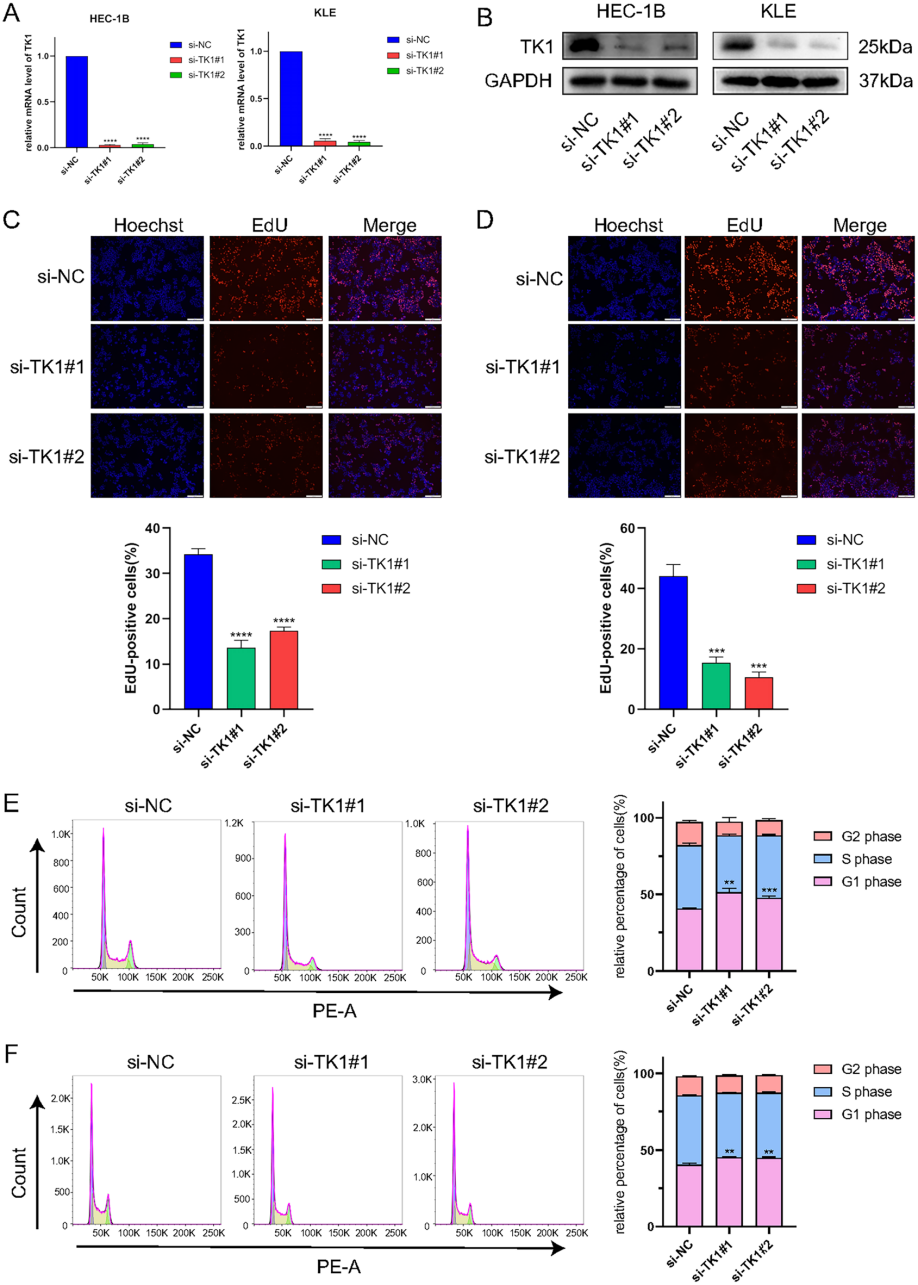
Experiment validation of cell proliferation and cell cycle after TK1 knockdown in vitro. (**A**) RT-qPCR and (**B**) western blot were used to detect the knockdown efficiency of TK1 in HEC-1B and KLE cell lines (blots were cut prior to hybridisation with antibodies during blotting). The proliferation capacities of UCEC cells were investigated by EdU assays in HEC-1B (**C**) and KLE (**D**). Cell cycle status was completed by flow cytometry in HEC-1B (**E**) and KLE (**F**).

After transfecting si-TK1 into HEC-1B and KLE, RT-qPCR and western blot were used to detect the knockdown efficiency (Fig. 7A,B). Both si-TK1#1 and si-TK1#2 showed great efficiency and were used to the following experiments. To evaluate the effect of TK1 on cell cycle, EdU assay was used and the results showed that the percentage of EdU-positive cells was significantly decreased in si-TK1#1 and si-TK1#2 group (Fig. 7C,D). Indicating the cell proliferative ability was decreased as well. In addition, flow cytometry was used to determine the DNA content in HEC-1B and KLE. Compared with si-NC, cells in si-TK1#1 and si-TK1#2 group were arrested in G1 phase (Fig. 7E,F), indicating that knockdown TK1 can inhibit cell cycle progression.

### Knockdown TK1 inhibited migration, invasion and EMT in UCEC cells

**Figure 8:**
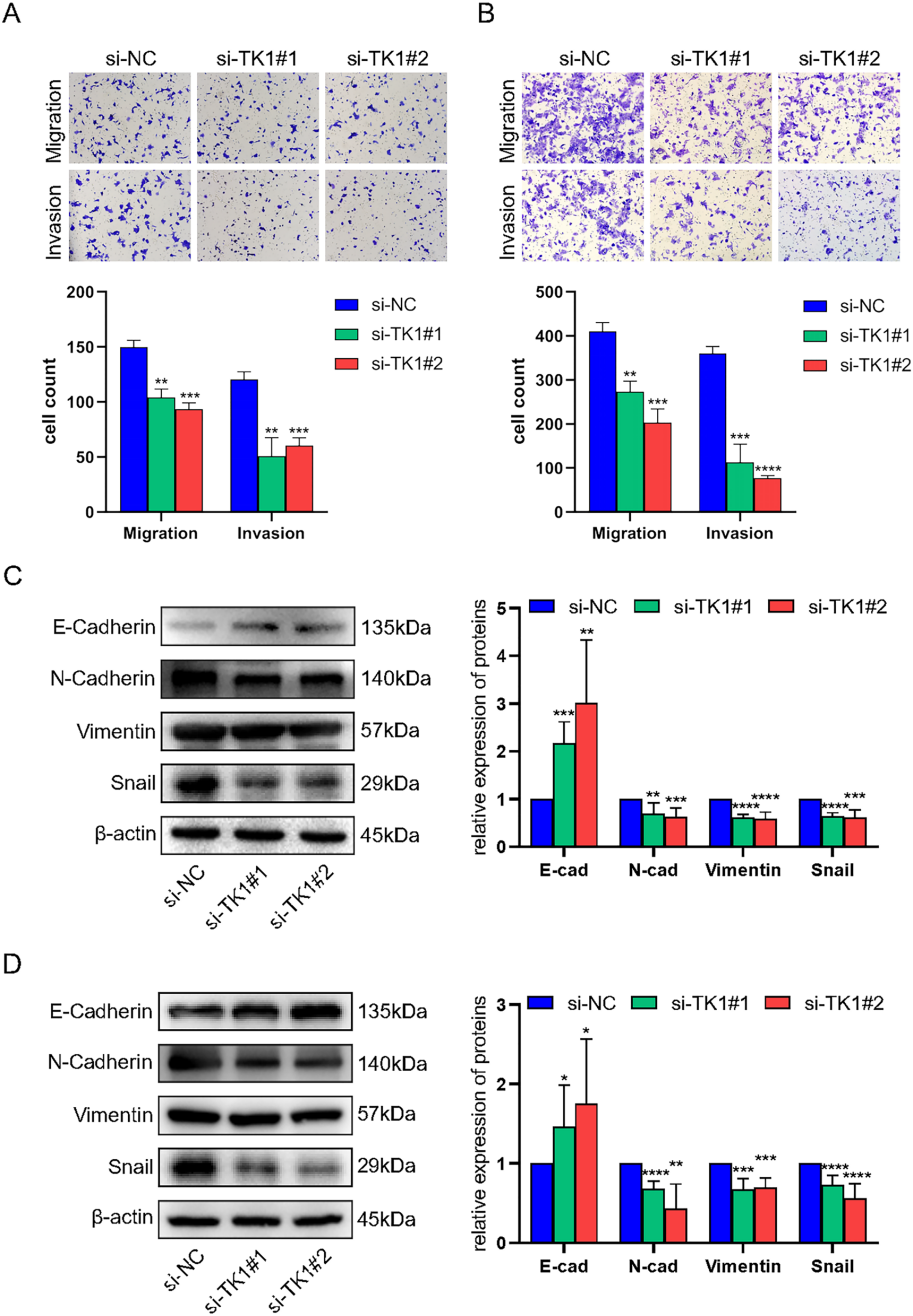
The migration, invasion and EMT in UCEC cells. Transwell assay was used to detect the ability of migration and invasion in HEC-1B (**A**) and KLE (**B**). Western blot was used to detect the EMT markers (blots were cut prior to hybridisation with antibodies during blotting) in HEC-1B (**C**) and KLE (**D**).

Except identifying the effect of TK1 on cell cycle, we also detected its influence on metastasis capacity of cells. Transwell system was used to estimate the ability of migration and invasion. As shown in the picture, TK1 knockdown could significantly inhibit the migration and invasion in HEC-1B and KLE (Fig. 8A,B). Epithelial-mesenchymal transition (EMT) is an important process in the development of tumorigenesis. In our results, compared to si-NC, TK1 knockdown could up-regulate the expression of E-cadherin and down-regulate the expression of N-cadherin, Vimentin and Snail (Fig. 8C,D).

## Discussion

In recent years, UCEC is the only major cancer increasing in both frequency and mortality. UCEC was usually divided into two types. Type I UCEC are related to estrogen excess and favorable prognosis, while type II UCEC are more in older women with a worse outcome^11^. According to the traditional pathological classification, correct subtyping of high-grade serous and endometrioid endometrial carcinoma is challenging^12^. Considering different treatment in endometrioid endometrial carcinoma and serous endometrial carcinoma, the new molecular typing provides more accurate suggestion for individuals. Thus, it’s important to find new target for molecular typing, diagnosis and prognosis.

Cell cycle regulators (including TK1) is critical for cell homeostasis. A primary cause of cancer is mutations or dysregulation of cell cycle proteins^3,13–15^. TK1 is an essential enzyme in DNA synthesis that is downstream of the CDK4/6 pathway. TK1 might be secreted from tumor cells that are growing actively. Elevated TK1 levels can be detected in both hematologic malignancies and solid tumors^16^. TK1 has been proved to be a new biomarker in several cancers, including hepatocellular carcinoma^17^, prostate cancer^18^, melanoma^19^, and breast cancer^20^ et al. In addition, TK1 combined with HE4 and CA125 showed a better diagnostic performance in ovarian cancer^21,22^. Serum TK1 can act as a novel biomarker in patients with HR(+)/HER2(−) advanced breast cancer^23^. Considering that TK1 has been less studied in UCEC, so we do this work to clarify the importance of TK1 in UCEC and conduct experimental validation.

As shown above, TK1 was overexpressed in UCEC compared with normal endometrial tissues. The clinical characteristics analysis revealed that TK1 was related to BMI, histological type, histological grade, clinical stage, and lymph node metastasis. High level of TK1 indicated a worse outcome in UCEC. TK1 expression was also correlated with UCEC molecular typing. Thus, TK1 might be a new biomarker for accurate classification and individual treatment. In addition, we also found that TK1 was strongly associated with cell cycle and DNA replication, indicating that abnormal expression of TK1 can promote the malignant behavior in UCEC. Based on the STRING and GeneMANIA, E2F1 might be a potential molecule that regulate TK1 expression. After E2F1 knockdown, TK1 expression decreased as well. ChIP assay revealed that E2F1 could combine with the promotor region of TK1 and regulate its expression. TK1 knockdown can significantly inhibit the cell proliferation ability and arrest the cell cycle in G1 phase. The results were consistent with TK1 function. As a DNA salvage pathway enzyme, TK1 participates in DNA synthesis and DNA damage. The concentration of TK1 is increasing at S phase and subsequently degradation by ubiquitination^24^. Moreover, TK1 can also inhibit the migration and invasion in vitro. EMT means a cellular process that cells lose the epithelial features and gain of mesenchymal features^25^. EMT has been linked to a number of tumor-related processes, including tumor development, malignant progress, tumor stemness and therapeutic resistance^25,26^. The common epithelial marker is E-cadherin and the mesenchymal markers include N-cadherin, Vimentin and Snail et al. Our results indicated that TK1 could also be a biomarker to evaluate tumor ability to metastasize.

Tumor microenvironment (TME) is crucial to tumorigenesis and composed with immune cells, cancer-associated fibroblasts, endothelial cells and et al^27^. Tumor infiltrating immune cells (TIICs) play a critical role in tumor proliferation and progression, either suppressive or supporting^27,28^. TK1 expression was negatively related to CD8 + T cell, macrophage and dendritic cell, indicating antigen presentation and lymphocyte killing dysfunction as well as immune escape probably occurred in UCEC. May be another reason for the poor prognosis of UCEC patients with high TK1 expression.

In conclusion, this study illustrates that TK1 may be a crucial biomarker for diagnosis and prognosis in UCEC. TK1 expression is also related to a supportive TME. TK1 knockdown can inhibit the proliferation, migration, invasion, EMT and results in cell cycle arrest in vitro.

## Methods

### Expression of TK1 in UCEC

Based on TCGA (V33.1, May 31, 2022, https://portal.gdc.cancer.gov/), we analyzed the TK1 expression in 26 cancer types and visualized by SangerBox (http://SangerBox.com/Tool). GEPIA (http://gepia.cancer-pku.cn/)^29^ was used to show the relationship of TK1 between UCEC tissues and normal tissues based on TCGA combined with GTEx cohort. In order to validate the TK1 expression in UCEC, GSE17025^30^ and GSE63678^31^ were taken from GEO database. The criteria to identify differential expression genes (DEG) was set with |log2FC|> 1 and *p* < 0.05. Besides, The Human Protein Atlas (V23.0, updates: June 19, 2023, https://www.proteinatlas.org/)^32^ was used to analyze TK1 in pathology altas. In addition, Kaplan–Meier Plotter database (updates: April 18, 2023, www.kmplot.com) was used to evaluate the TK1 expression in prognostic value.

### Clinical value of TK1 in UCEC

To identify the relationship between TK1 and clinical characteristic features in patients with UCEC, we acquired clinical information of UCEC from TCGA. Chi-square test was used to analyze the association between TK1 and clinicopathological parameters.

### DNA methylation of TK1 in UCEC

UALCAN (updates: August 16, 2022, https://ualcan.path.uab.edu/index.html) database^33^, MethSurv database (https://biit.cs.ut.ee/methsurv/)^34^ and SMART (http://www.bioinfo-zs.com/smartapp/) website^35^ were used to evaluated the DNA methylation of TK1 in UCEC based on TCGA.

### Gene alteration of TK1 in UCEC

Analysis of TK1 genetic alterations was performed with cBioPortal database (updates: May 2, 2023, https://www.cbioportal.org/)^36^. To better clarify the mutation type and base mutation of TK1 in UCEC, COSMIC (https://cancer.sanger.ac.uk) was used with the mode of “tissue distribution” and “mutation distribution” in endometrium. In addition, we analyzed other genes mutations based on TK1 expression.

### Protein interactions of TK1 in UCEC

STRING (V11.5, updates: August 12, 2021, www.string-db.org)^37^ and GeneMANIA (V3.5.2, updates: February 26, 2020, www.genemania.org)^38^ website were used to build the network between TK1 and related proteins. In these two databases, the species was set with “Homo sapiens”. In STRING, we set minimum required interaction score at high confidence (0.700), max number of interactors to show 20 interactions. In GeneMANIA, physical interactions, co-expression, predicted, co-localization, genetic interactions, pathway and shared protein domain were selected to show in the result.

### Gene enrichment analysis

LinkedOmics (updates: October 8, 2018, http://www.linkedomics.org/login.php)^39^ was used on UCEC to discover critical genes associated with TK1. GO and KEGG pathway enrichment analysis were performed using R package ‘clusterProfiler’ to further explore the function of TK1 related genes.

### Immune infiltration analysis

TIMER2.0 (V2.0, https://cistrome.shinyapps.io/timer/)^40^ was used to evaluate the link between TK1 level and immune cell infiltration in UCEC. In addition, TISDIB (http://cis.hku.hk/TISIDB/) database^41^ was applied to explore the relationship between TK1 expression and immune cell infiltration abundance in UCEC.

### siRNA(shRNA) transfection

Small interfering RNA (siRNA) and short hairpin RNA (shRNA) were obtained from Shanghai GenePharma Co., Ltd for TK1 and E2F1 knockdown. HEC-1B and KLE were plated into 6-well plates before transfection. 5 μl si-TK1 and 5 μl Lipofectamine 3000 (Thermo Fisher Scientific, Inc.) were used to perform the procedure according the instruction. 48 h later, cells were harvested to complete other experiments. 1 μl sh-E2F1 and 2 μl polybrene were used to complete sh-E2F1 transfection into HEC-1B. 72 h later, HEC-1B was harvested to detect E2F1 expression and ChIP assay. siRNA(shRNA) oligos sequences are listed in supplementary table S1.

### RT-qPCR

Total RNA was extracted using TRIzol (15596026, Thermo Fisher Scientific) reagent. cDNA was synthesized via a reverse transcription kit (00984912, Thermo Fisher Scientific). RT-qPCR was performed using the SYBR Green reaction mix (B21703, Bimake). QuantStudio 3 (Thermo Fisher Scientific) was used to complete the reaction. 2^−ΔΔCt^ method was used to analyze the results of RT-qPCR. The primer sequences are listed in supplementary tables S2–S3.

### ChIP assay

ChIP assay was completed using SimpleChIP Plus Sonication Chromatin IP Kit (56383, CST). The samples were prepared through cross-linking, chromatin fragmentation, chromatin immunoprecipitation, elution of chromatin from antibody/protein G magnetic beads and reversal of cross-links and DNA purification. Finally, the DNA samples were quantified by RT-qPCR.

### EdU assay

Five thousand HEC-1B and KLE were plated into 96-well plates 24 h after siRNA transfection. After 48 h, the procedure was performed according to the instructions of EdU kit (Cat. K1077, APExBIO). The images were captured by a fluorescence microscopy.

### Transwell assay

To assess the ability of migration and invasion, 8 μm transwell chambers (3422, Corning) were placed in 24-well plates. 10^5^ cells in 200 μl serum-free media were plate in the upper chamber, while 20% fetal bovine serum was added to the culture in the lower chamber. The diluted matrix gel (354248, Corning) (matrix gel:serum-free media = 1:7) should be placed into the upper chamber an hour before the invasion experiment begins. The cells were fixed with 4% paraformaldehyde and stained with 0.1% crystal violet after 48 h. Transwell chambers were observed and photographed with an upright microscope, and 5 fields of view were randomly selected for cell counting by eyes.

### Western blot

Cells in plates were collected and lysed using RIPA added PMSF (0010, Solarbio) (RIPA:PMSF = 100:1). After centrifuge, a BCA protein assay kit was used to detect the protein concentration. SDS-PAGE was used to perform the experiment with 30 μg protein samples. Then transferred the protein to the PVDF membrane (10600023, Cytiva) and blocked with 5% skim milk. Incubated the PVDF membrane overnight at 4 °C with primary antibodies then added secondary antibodies conjugated with horseradish peroxidase. Finally, images were detected using a gel imaging system. Antibodies used in western blot are listed in supplementary table S4. Membranes were cut according to the molecular weight of the target protein prior to hybridisation with antibodies and then exposed it. Original blots are presented in supplementary file 2.

### Cell cycle assays

Cells were collected and fixed with 70% alcohol overnight at 4 °C. Cell cycle assays were performed by DNA Content Quantitation Assay (CA1510, Solarbio). Finally, cells were analyzed using BD FETASSA.

### Statistical analysis

GraphPad Prism 8.0 was used to do all statistical analyses. Two experimental groups were compared using the student's t-test. The figures' error bars show the mean ± the standard deviation (SD). *p < 0.05, **p < 0.01, ***p < 0.001, ****p < 0.0001, and ns = not significant were the criteria used to determine significance. Every outcome was verified at least three separate times.

### Supplementary Information

Supplementary Information 1.

Supplementary Information 2.

## Data Availability

The datasets used and analyzed during the current study are available from the corresponding author on reasonable request.
