# Author Correction: A comprehensive analysis and experimental validation of TK1 in uterine corpus endometrial carcinoma

**DOI:** 10.1038/s41598-024-59542-1

**Published:** 2024-04-17

**Authors:** Yiqing Sun, Kaiwen Zhang, Tianqi Wang, Shuangshuang Zhao, Chao Gao, Fengxia Xue, Yingmei Wang

**Affiliations:** 1 Department of Gynecology and Obstetrics, Tianjin Medical University General Hospital, Tianjin, 300052 China; 2 Tianjin Key Laboratory of Female Reproductive Health and Eugenics, Tianjin Medical University General Hospital, Tianjin, 300052 China

Correction to: *Scientific Reports* 10.1038/s41598-024-56676-0, published online 13 March 2024

The original version of this Article contained an error in Figure 7B. Due to errors during figure assembly, the western blot panel for ‘KLE’ ‘TK1’ showed a different replicate of the same experiment that was not run on the same membrane as the depicted ‘GAPDH’ western blot. Consequently, the original ‘TK1’ blot was replaced with the ‘TK1’ blot belonging to the corresponding ‘GAPDH’ blot.

The original Figure 7 appears below.

**Figure 7:**
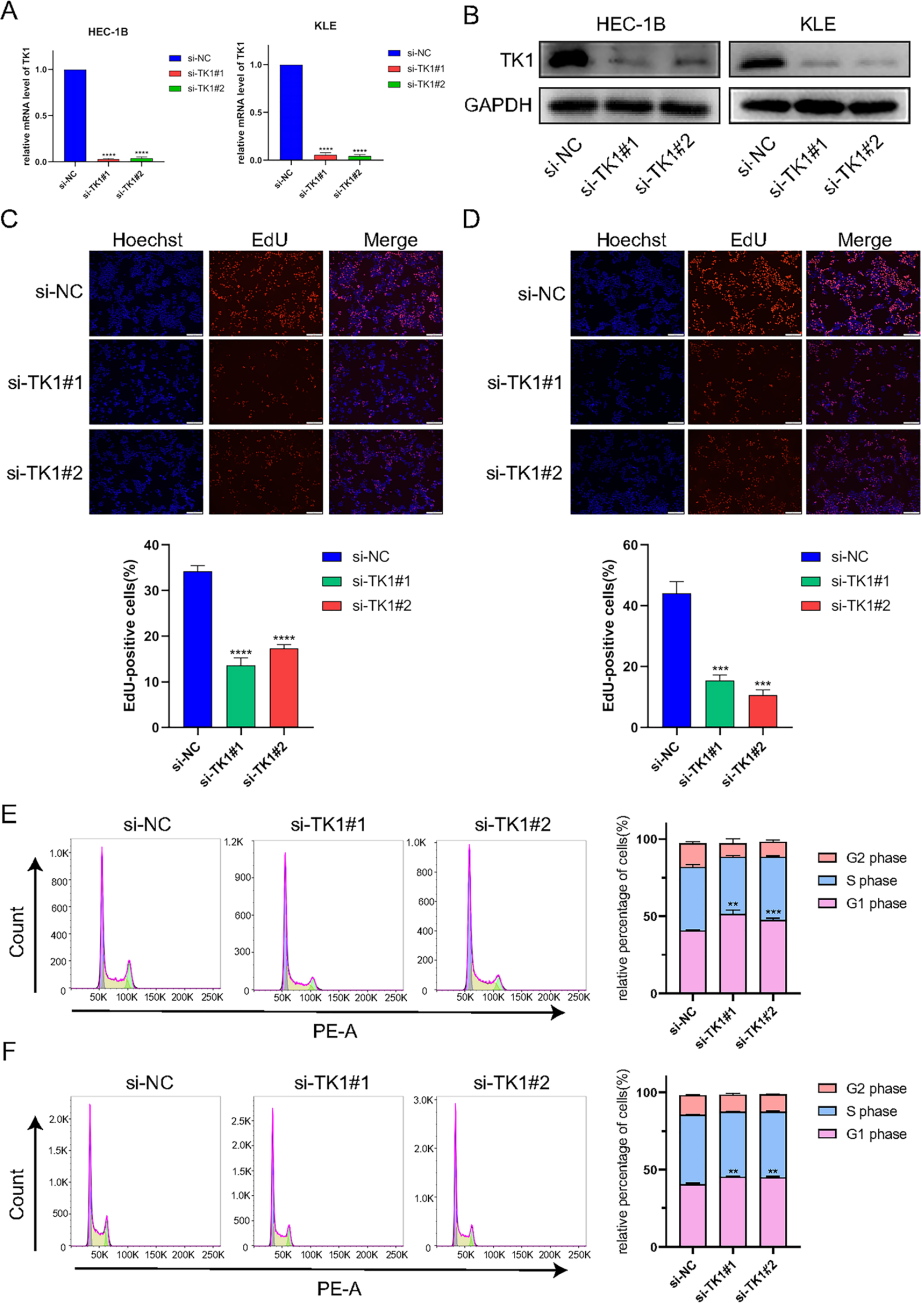
Experiment validation of cell proliferation and cell cycle after TK1 knockdown in vitro. (**A**) RT-qPCR and (**B**) western blot were used to detect the knockdown efficiency of TK1 in HEC-1B and KLE cell lines (blots were cut prior to hybridisation with antibodies during blotting). The proliferation capacities of UCEC cells were investigated by EdU assays in HEC-1B (**C**) and KLE (**D**). Cell cycle status was completed by flow cytometry in HEC-1B (**E**) and KLE (**F**).

The original Article has been corrected.

