# Supplementary material for: A comprehensive analysis and experimental validation of TK1 in uterine corpus endometrial carcinoma
Source: Sci Rep. 2024 Mar 13;14:6134. doi: 10.1038/s41598-024-56676-0 (PMC10937635; doi:10.1038/s41598-024-56676-0)
Supplement: Supplementary file 1 — Supplementary Information 1. [file 41598_2024_56676_MOESM1_ESM.docx]

A comprehensive analysis and experimental validation of TK1 in Uterine Corpus Endometrial Carcinoma


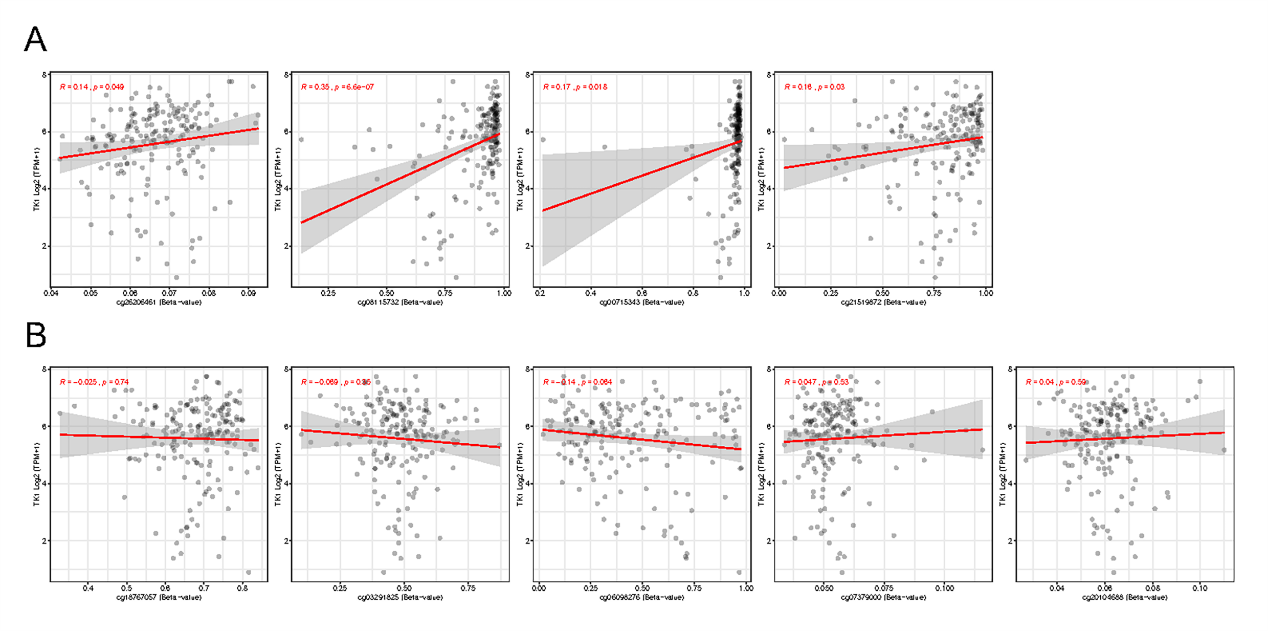


**Supplementary Figure 1.** The relationship between methylation sites and TK1 expression. (A)The methylation sites positively related to TK1 expression. (B) The methylation sites not significantly related to TK1 expression.

**Supplementary Table S1.**

| **siRNA(shRNA) oligos** |  |
| --- | --- |
| si-TK1#1-F | GCAUUAACCUGCCCACUGUTT |
| si-TK1#1-R | ACAGUGGGCAGGUUAAUGCTT |
| si-TK1#2-F | GGUGAUCAAGUAUGCCAAATT |
| si-TK1#2-R | UUUGGCAUACUUGAUCACCTT |
| sh-E2F1#1-F | GGACUCUUCGGAGAACUUUTT |
| sh-E2F1#1-R | AAAGUUCUCCGAAGAGUCCTT |
| sh-E2F1#2-F | GCUGGACCACCUGAUGAAUTT |
| sh-E2F1#2-R | AUUCAUCAGGUGGUCCAGCTT |

**Supplementary Table S2.**

| **qRT-PCR Primers** |  | **Product length** |
| --- | --- | --- |
| GAPDH-F | GTCTCCTCTGACTTCAACAGCG | 131 |
| GAPDH-R | ACCACCCTGTTGCTGTAGCCAA |  |
| TK1-F | AGCAGCTTCTGCACACATGACC | 144 |
| TK1-R | CTCGCAGAACTCCACGATGTCA |  |

**Supplementary Table S3.**

| **ChIP-qPCR Primers** |  | **Product length** |
| --- | --- | --- |
| E2F1-chip-F | TGGAAATCAGGGCGATGGAC | 156 |
| E2F1-chip-R | TCCATCATGGCGTCTACAGC |  |

**Supplementary Table S4.**

| **Name** | **Company;**  **Catalog numbers** | **Antibody dilutions** |
| --- | --- | --- |
| GAPDH | CST; 5174 | 1:1000 |
| β-Actin | CST; 3700 | 1:1000 |
| E2F1 | CST; 3742 | 1:1000 |
| E-cadherin | CST; 3195 | 1:1000 |
| N-cadherin | CST; 13116 | 1:1000 |
| Vimentin | CST; 5741 | 1:1000 |
| Snail | CST; 3879 | 1:1000 |
| TK1 | Proteintech; 67787-1-Ig | 1:2000 |
