# Supplementary material for: A comprehensive analysis and experimental validation of TK1 in uterine corpus endometrial carcinoma
Source: Sci Rep. 2024 Mar 13;14:6134. doi: 10.1038/s41598-024-56676-0 (PMC10937635; doi:10.1038/s41598-024-56676-0)

Membranes were cut according to the molecular weight of the target protein prior to hybridization with antibodies and then exposed (we also explained this in the methods on page 8, lines 16-18 and figure legends). In this file, we provided membranes with visible edges and three replicates. Additionally, we added molecular size markings with the blots. In order to better match these images to the figures in the manuscript, we denoted the regions of the original blots used in main figures using red boxes. All digitised images submitted with the final revision of the manuscript are 300 DPI.

Figure 4E

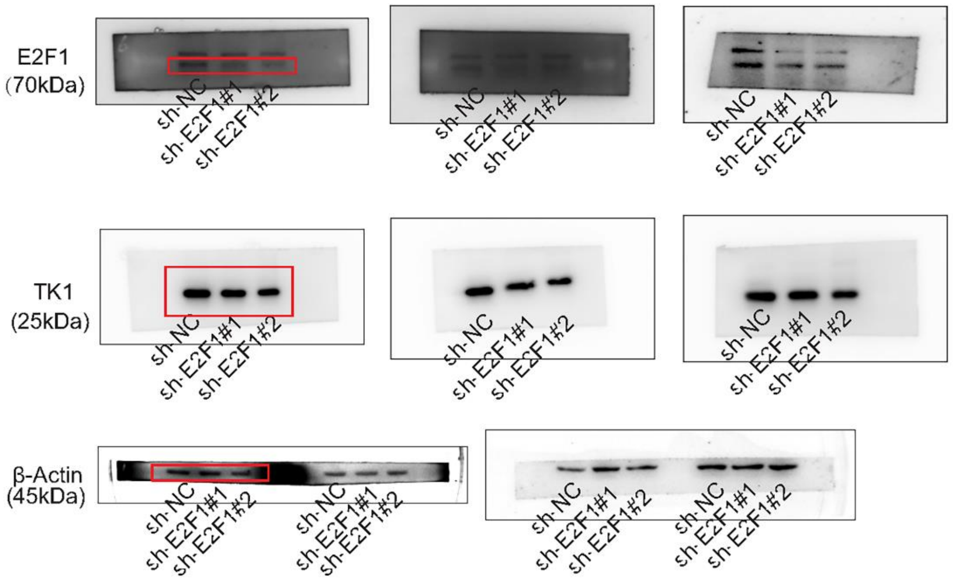

Figure 7B (HEC-1B)

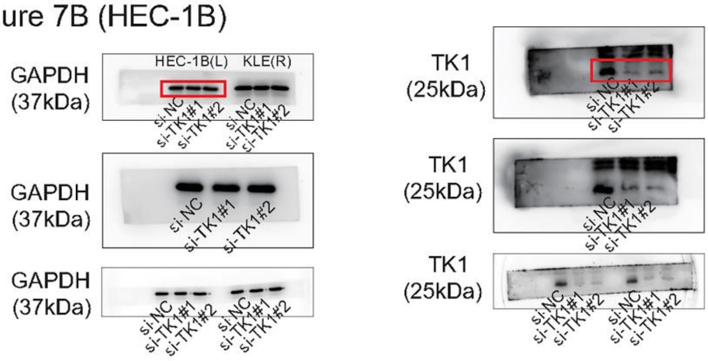


Figure 8C (HEC-1B)

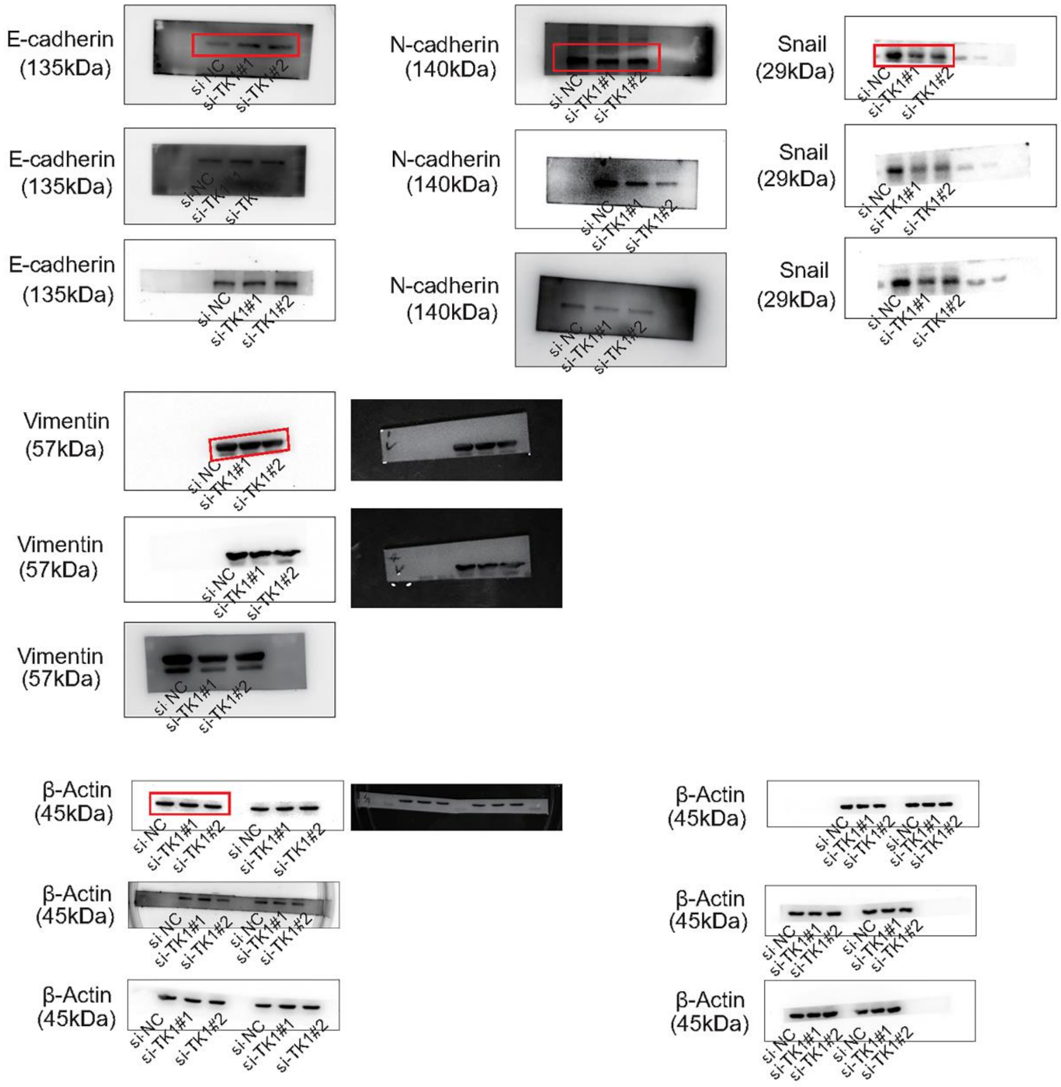

Figure 7B (KLE)

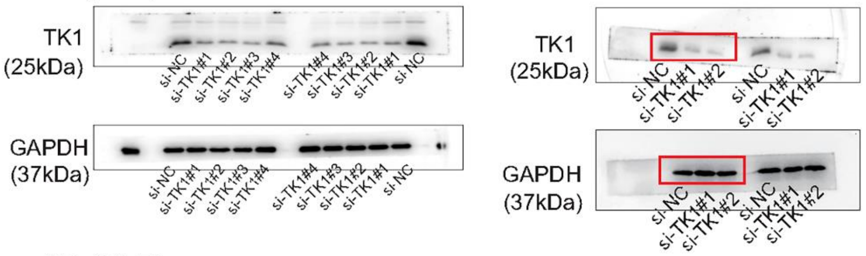


Figure 8D (KLE)

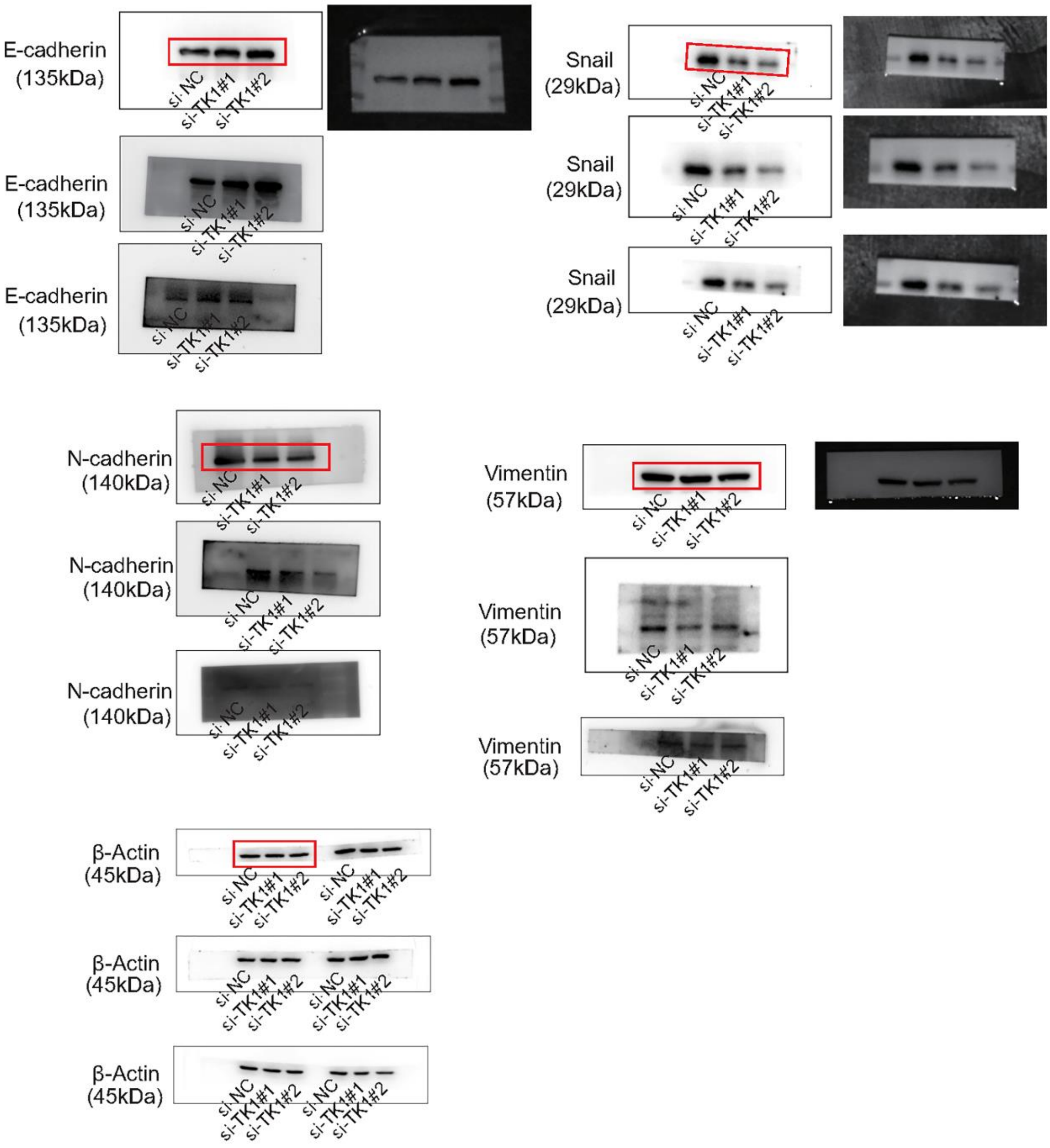

Supplement: Supplementary file 2 — Supplementary Information 2. [file 41598_2024_56676_MOESM2_ESM.pdf]
